# Implementation of HIV Self-Testing to Reach Men in Rural uMkhanyakude, KwaZulu-Natal, South Africa. a DO-ART Trial Sub Study

**DOI:** 10.3389/fpubh.2021.652887

**Published:** 2021-08-03

**Authors:** Nsika Sithole, Maryam Shahmanesh, Olivier Koole, Meighan Krows, Torin Schaafsma, Mark J. Siedner, Connie Celum, Ruanne V. Barnabas, Adrienne E. Shapiro

**Affiliations:** ^1^Clinical Research Department, Africa Health Research Institute, Somkhele, South Africa; ^2^Institute for Global Health, University College London, London, United Kingdom; ^3^London School of Hygiene and Tropical Medicine, London, United Kingdom; ^4^Department of Global Health, University of Washington, Seattle, WA, United States; ^5^Division of Infectious Diseases, Massachusetts General Hospital, Boston, MA, United States; ^6^Harvard Medical School, Boston, MA, United States; ^7^Department of Epidemiology, University of Washington, Seattle, WA, United States; ^8^Division of Infectious Diseases, Department of Medicine, University of Washington, Seattle, WA, United States

**Keywords:** self testing, HIV infection, men, South Africa, mass screening

## Abstract

**Background:** KwaZulu–Natal, South Africa has one of the highest HIV prevalence rates globally. Persons <35 years and men have lower rates of HIV testing. HIV self-testing (HIVST) may overcome many barriers of facility-based HIV testing in order to identify HIV positive young persons and men and link them to care. We investigated whether HIVST distribution was a feasible approach to reach men and assessed the proportion of participants who reported their HIVST results, tested positive and linked to care.

**Methods:** Teams comprised of a nurse, clinic research assistant, and recruiters distributed HIVST kits in rural uMkhanyakude, KwaZulu-Natal from August—November 2018 with a focus on testing men. Workplaces (farms), social venues, taxi ranks, and homesteads were used as HIVST kit distribution points following community sensitisation through community advisory boards and community leaders. HIVST kits, demonstration of use, and small incentives to report testing outcomes were provided. The Department of Health provided confirmatory testing and HIV care at clinics.

**Results:** Over 11 weeks in late 2018, we distributed 2,634 HIVST kits of which 2,113 (80%) were distributed to persons aged <35 years, 2,591 (98%) to men and 356 (14%) to first time testers. Of the HIVST distributed, 2,107 (80%) reported their results to the study team, and 157 (7%) tested positive. Of persons who tested positive, 107/130 (82%) reported having a confirmatory test of which 102/107 (95%) were positive and initiated on ART. No emergencies or social harms were reported.

**Conclusion:** Large scale distribution of HIVST kits targeting men in rural KwaZulu-Natal is feasible and highly effective in reaching men, including those who had not previously tested for HIV. While two-thirds of persons who tested HIV positive initiated ART, additional linkage strategies are needed for those who do not link after HIVST. HIVST should be used as a tool to reach men in order to achieve 95% coverage in the UNAIDS testing and care cascade in KwaZulu-Natal.

## Introduction

HIV-associated mortality continues to be high among persons who have barriers to accessing routine health services, particularly African men (1–6). The risk of death to HIV positive persons not engaged in care is 10 times higher than that of HIV negative persons (5). In many settings in sub-Saharan Africa, men are less likely to test for HIV, HIV-positive men are less likely to link to HIV clinical services and start ART at lower rates than women, are more likely to be lost to care, and more likely to die at every stage (1–6). HIV treatment coverage is higher among South African women than men, with 65% of adult women living with HIV on treatment, compared to 56% of adult men (7). Even where an equal proportion of men and women are found to make use of HIV testing services (HTS), men are more likely to get tested for HIV after progressing to advanced disease (8). Data from the South African Demographic and Health Survey suggest that men in general access health services less readily than women (9). Barriers for South African men to access health services arise from multiple factors, including stigma, preference for traditional medicine, cultural ideals of masculinity, and practical issues including an inconvenience with the clinic operating times and problems with transportation (10, 11). KwaZulu-Natal Province has the highest HIV prevalence [27.4% (95% CI: 25.9–28.9%) in 2018] and incidence [1.17% (95% CI: 0.99–1.35%) in 2018] for those aged 15–49 years in South Africa (12, 13) and in this province, individuals aged <35 years and men account for most of the people untested for HIV (14).

Since men are less likely to attend standard facility-based services for testing, more convenient and different testing strategies such as HIV self-testing (HIVST) are needed in order to achieve the “first 95” (95% of persons with HIV knowing their status) in the UNAIDS 95-95-95 testing and care cascade in rural KwaZulu Natal, South Africa. HIVST, using a simple oral-fluid or blood-based self-test at a time and place convenient to the person testing, could overcome some barriers that deter people from testing (15). Furthermore, HIVST may be more convenient for users as it displays the potential to reduce the number of facility visits for frequent testers and eliminate the need for individuals to travel long distances or wait in long lines to access HIV testing (16). The World Health Organization (WHO) has proposed HIVST as an approach to reach people who are not accessing existing HTS such as men and young people (17).

The Delivery Optimization of Antiretroviral Therapy (DO ART) study was developed in part to address disparities in access to HIV care for men (18). In order to further provide access to men, we implemented the HIVST programme. We conducted the programme in uMkhanyakude district in northern KwaZulu-Natal, South Africa with the objectives of providing HIVST as an alternative testing strategy to clinic-based testing, investigating whether HIVST distribution was acceptable in the community, and determining its feasibility to reach men to improve access for testing. In addition to assessing HIV self testing uptake among men, we evaluated the proportion of participants who reported their HIVST results, tested positive and linked to care in both short and long term intervals.

## Methods

The HIVST programme was developed as a sub-study of the DO ART study, which compared community-based ART delivery to standard clinic-based ART services for people with HIV newly initiating on ART (18). One of DO ART's objectives was to determine whether community-based services improved HIV testing and outcomes in men; and initially, clinic-based recruitment identified few male participants since clinic attendance by men is low. Community-based recruitment yielded a greater amount of men than clinic–based recruitment but HIVST would simplify testing, include the opportunity to test privately, and potentially increase the coverage of testing among men. We introduced HIVST as a strategy to increase HIV testing among men and to identify men who would benefit from ART initiation. In August 2018, when the HIVST sub- study was implemented, 47% (*n* = 99) of DO ART enrolees in the uMkhanyakude district site were men.

### Procedures

#### Identifying Men for HIVST

During the distribution period (August–November 2018), three teams of a nurse and clinical research assistant and four recruiters canvassed the district to introduce and distribute HIVST kits to men as an alternative way to test for HIV. Each team of a nurse and clinic research assistant typically spent afternoons on HIVST kit distribution, while the 4 recruiters spent the day on identifying eligible men for kit distribution.

**Figure 1:**
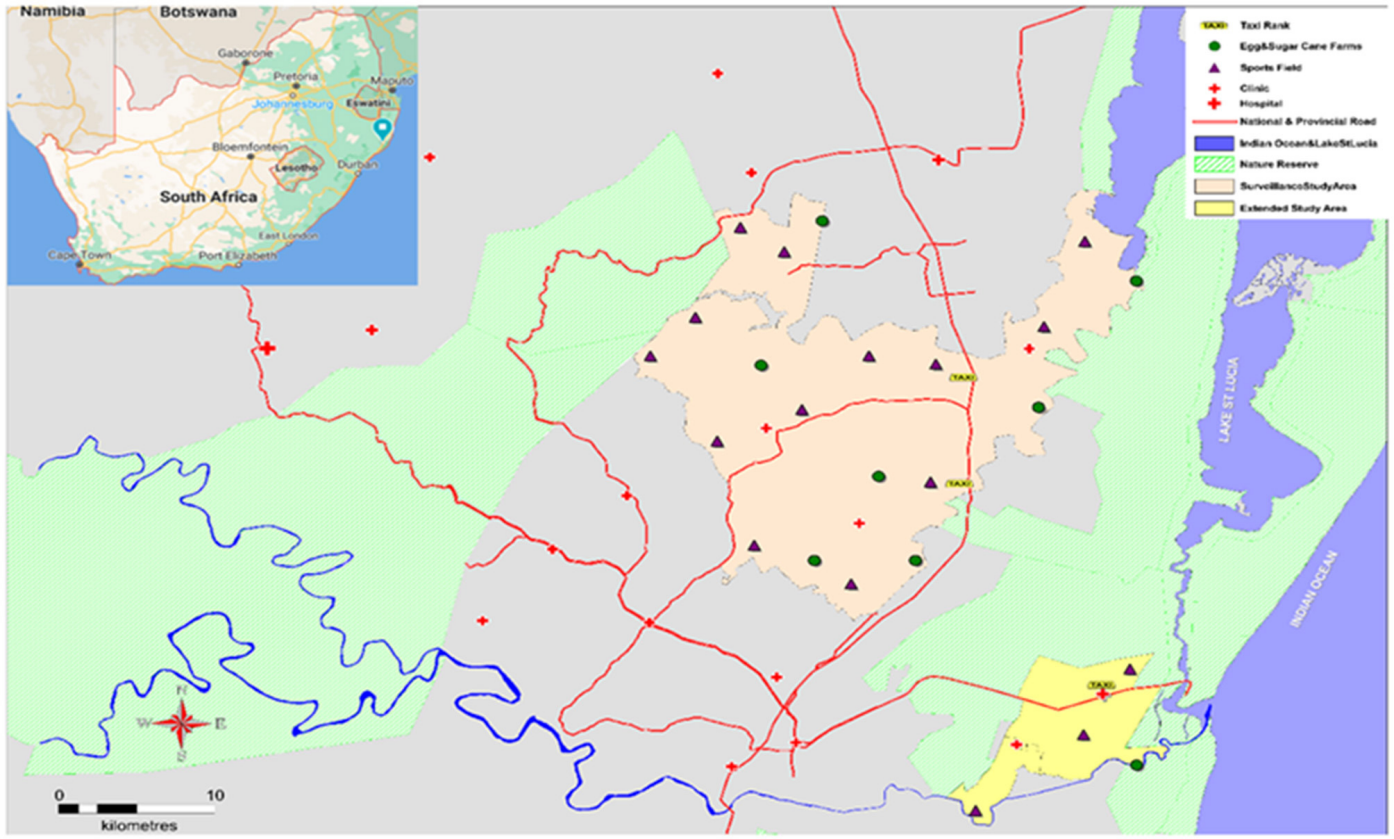
Study area with HIVST kit distribution points.

The Africa Health Research Institute (AHRI) has a long-standing demographic surveillance platform in much of the Hlabisa sub-district (Figure 1) of uMkhanyakude, which allowed the study team to enter and distribute kits within these sites based on our existing relationship with the communities. We extended recruitment and kit distribution beyond these areas by approaching the local chiefs and obtaining permission to conduct study activities in their regions.

The recruitment teams identified locations in the district where men were found to congregate, like workplaces (commercial farms), social venues such as sports grounds, taxi ranks, and the streets. Kit distribution was operationalised in phases. Workplace distribution entailed liaising with farm managers for permission to distribute kits on-site. The farm managers gave us permission as long as kit distribution did not interfere with the farm workers' daily duties. We scheduled kit distribution accordingly and found that Sundays and late afternoons were the best times to distribute in the farms. The second phase of kit distribution established community settings where the recruitment teams identified areas where men who are unemployed congregate. This was the more successful phase as we were able to find men in larger numbers in these areas as unemployment is at 42% in uMkhanyakude district (19). The area was divided amongst the team with each team or recruiter being assigned to a specific section of the area. The team focused on recruitment in their assigned area until all sections of the area had been covered.

#### HIVST kit Distribution Procedures

Procedures for each HIVST kit distribution event have been previously described (20). Briefly, at each recruitment point, participants 18 years and older were given an option to choose between an oral fluid-based HIVST or a finger prick blood-based HIVST. Study team members conducted a live demonstration of how to use and interpret both test kits, and provided cell-phone videos demonstrating kit use, available for watching on site or at home. Study team members also provided counseling that HIVST kits should not be used for testing if persons already knew they had HIV and that HIVST kits may be inaccurate if used in persons taking ART. Written informed consent was obtained prior to kit distribution.

Participants had the option of doing on-site testing using the kit in a private booth, with or without the assistance of a staff member, or choose the option of taking the kit away from the testing site and testing later. Before being issued a test kit, participants completed a questionnaire which included questions on demographics, HIV testing history, sexual behaviors, alcohol use, test kit preferences and cell phone number. Data was collected using Mobenzi software (Cape Town, South Africa) administered on a Samsung smartphone. All who took HIVST kits received a test kit labeled with a unique study identification number. After completing the test, participants were asked to report their results to the study team. All participants received a call back card with their HIVST kit to report the results of their self-test to the study team, as well as a cell phone airtime voucher (valued at USD2) to be redeemed at the time of reporting results. Persons taking kits off site reported results by calling or sending an SMS/text message to the number on the call back card for a free call back by the study staff who issued the self- test kit. Staff then contacted the participant and asked for the HIVST result. Staff provided post-HIVST counseling (in person if on-site or over the phone if off-site) including referral for confirmatory testing and ART initiation if the HIVST was confirmed to be reactive, or HIV prevention information and referral for voluntary male medical circumcision if the HIVST was non-reactive. Participants received a non-identifying reminder text message after 2 weeks if they had not yet reported their result. (“Act now test for HIV! Did the test? Call or send a Please Call Me to XXX”). The reminder was repeated at 1 and 2 months after distribution. If no result was reported by 2 months, staff made an outreach call to assess test use and results. After participants reported their results and received counseling and referrals, staff distributed the airtime incentive and administered a brief questionnaire to assess experience, usability, acceptability and preferences about HIVST.

#### Linkage to Care and ART for HIV Positive Men

HIVST distribution was done in partnership with the Department of Health (DOH). The DOH agreed to provide confirmatory HIV testing and linkage to ART initiation at their clinic facilities. In order to address concerns that persons testing positive on their own using HIVST, may experience social harms or personal stress in the absence of having face-to-face post-test counseling available, the HIVST programme provided all participants who took an HIVST kit with a 24-h mobile number which they could use if they felt the need to speak to a health care professional, and created procedures for post-test counseling to be provided telephonically when the participants were reporting their test results to the study teams.

Linkage to care was also offered through the DO ART study (18). Participants who reported a positive HIVST could elect to be visited by study staff members who offered to do confirmatory testing using rapid tests. Those who were confirmed to be positive were offered a chance to be screened for DO ART.

Some participants who reported positive HIVST results were not able to be contacted by study staff to assess linkage to care due to cell phone numbers no longer working or participants emigrating out of the area for employment opportunities. We reviewed individual records for each of these participants using national databases to identify any evidence of linkage to care through DOH clinics.

#### Long Term Analysis of Linkage to Care (15 Months After the end of the Follow up Period)

In September 2020, we conducted a long-term follow-up analysis using the South African National Health Laboratory Services (NHLS) database to identify if participants who tested positive but had not linked to care by the end of the follow-up period had subsequently linked to care. Documented HIV viral load and CD4 results in the database were used as evidence of linkage.

### Statistical Analysis

Percentages were used for descriptive analysis. We fit univariate and multivariate regression models in R (version 4.0) to identify predictors for those who reported back their HIVST results, those who tested positive and those who successfully linked to care during study follow-up. Odds ratios with *p* < 0.05 were considered statistically significant.

### Ethical Considerations

Ethical approval was obtained from the University of Washington, Human Sciences Research Council and the University of KwaZulu-Natal Ethics Committees.

## Results

In a 11 week interval between August and November 2018, a team consisting of 10 staff members distributed 2,634 HIVST kits to South African adults in rural uMkhanyakude district KwaZulu-Natal. Men were the recipients of [2,591(98%)] kits. Among the men who participated in HIVST, 44% were unemployed while almost a third (31%) reported they were laborers/semi-skilled. Three hundred and fifty-six (14%) of men were first-time HIV testers. From the kits that were distributed, [2,113(80%)] kits were distributed to participants aged <35 years.

The majority of those who received kits [2,294(87%)] preferred to take the kits off-site instead of using the HIVST kit on-site (Table 1). Most of the men [2,558(97%)] were unmarried with one or more current sexual partner and the median age was 27 years (IQR 22 to 33). Almost half [1,266(49%)] of the men were circumcised and [1,511(68%)] reported alcohol use. A total of [1,624(62%)] participants preferred to use the blood-based kits, while [1,010(38%)] selected to use the oral fluid kits. From those who took kits off site, [933(35%)] preferred to use oral fluid kits and [1,361(52%)] preferred blood-based kits. A total of [2,258(86%)] reported that their last HIV test was negative. From the 356 first time testers, [35(10%)] were found to have tested positive through HIVST.

**Table 1:** Characteristics of HIVST kit recipients.

		***N***	**(%)**
Distribution setting	Mobile van	2,344	(89%)
	Venue-based	154	(6%)
	Work place	86	(3%)
	Other	5 0	(2%)
Kit type	OraQuick (Oral based)	1,004	(38%)
	Atomo (I-test)	1,630	(62%)
On site testing	OraQuick	71	(3%)
	Atomo (I-test)	269	(10%)
Off site testing	OraQuick	933	(35%)
	Atomo (I-test)	1,361	(52%)
Age, median (IQR)	27 (22-33)		
Gender	Male	2,591	(98%)
	Female	43	(2%)
Education	Primary	387/2,618	(15%)
	Secondary and above	2,231/2,618	(85%)
Marital Status	Married	76	(3%)
	Not married	2,558	(97%)
Employment status	Laborer/semi-skilled	812	(31%)
	Unemployed	1,157	(44%)
	Student	367	(14%)
	Other	297	(11%)
Number of current sex partners	1	1,368/2,623	(52%)
	0	90/2,623	(3%)
	2+	1,165/2,623	(44%)
Circumcised		1,266/2,584	(49%)
Alcohol use (drinks in past week)	0	1,100/2,611	(42%)
	1–6	1,208/2,611	(46%)
	7+	303/2,611	(12%)
Ever tested for HIV	Yes	2,270	(86.4%)
	No	1,208/2,611	(13.5%)
	N/A	8	(0.3%)
Last HIV test	More than a year ago	1,002	(38%)
	Within 12 months	1,203	(45.7%)
	N/A	429	(16.3%)
Latest test result	Negative	2,258	(85.7%)
	Positive	7	(0.3%)
	Didn't receive the results	1	(0.0%)
	N/A	368	(14%)

*IQR, inter-quartile range, Other includes professional, farming, housewife and trade/sales categories*.

**Figure 2:**
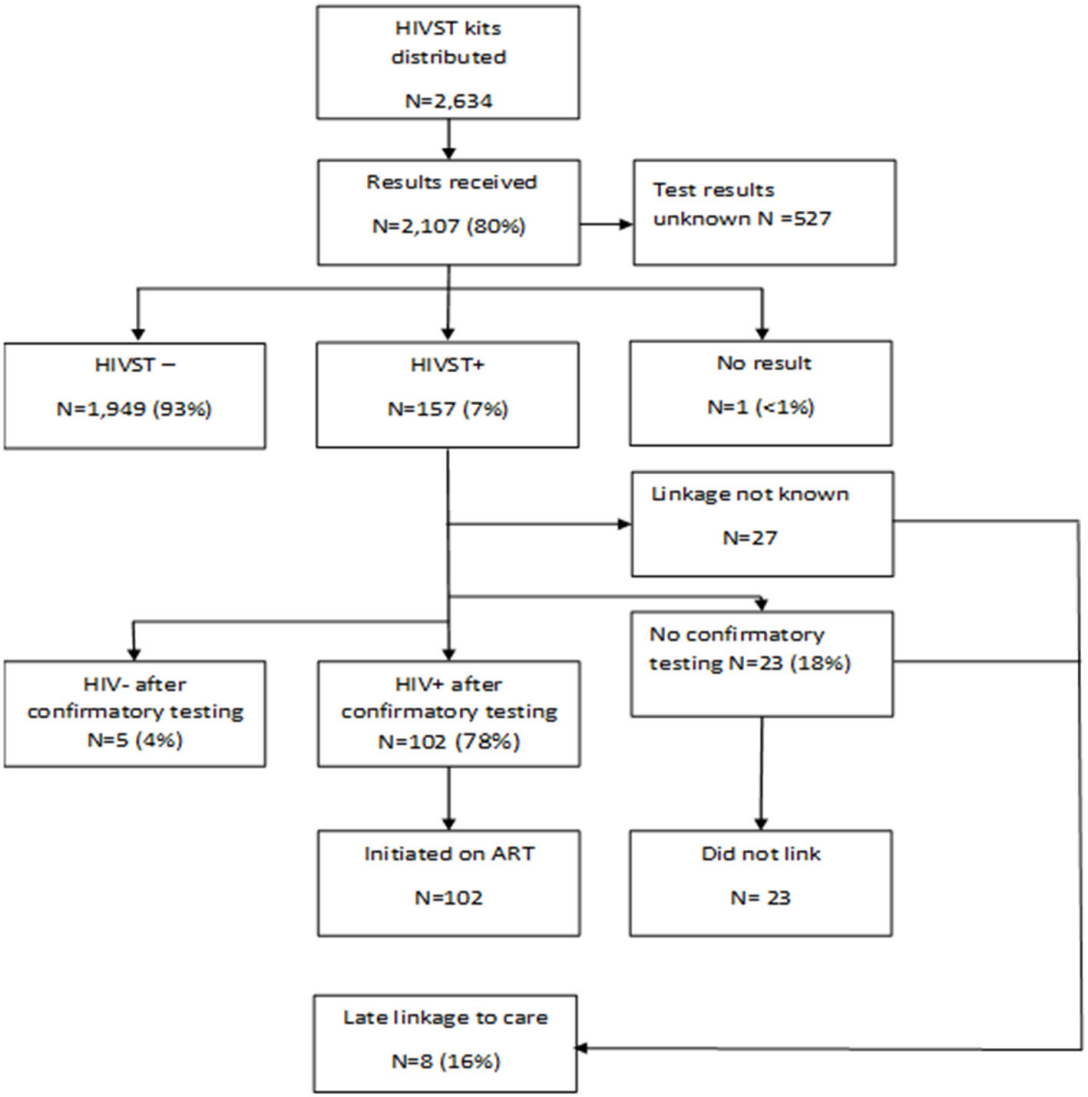
HIVST testing, results and linkage to care flow chart. Percentages are relative to the pervious linked box(es).

A total of [2,107(80%)] participants used the HIVST kits and reported their results to the study team. Among persons who reported their results, [157(7%)] tested positive and [102(65%)] were confirmed to have linked to care (Figure 2). There were 5 participants who reported a positive HIVST result but subsequently had HIV-negative confirmatory testing, indicating a false-positive HIVST for a total of 152 true positives. Twenty-three men who reported a positive HIVST result had not sought confirmatory tests and did not link to care for ART initiation by the end of June 2019, which was the end of the follow-up period. An additional 27 men had unknown linkage history after they initially reported testing positive through HIVST. No emergencies were reported on the 24-h cell phone number.

We evaluated predictors of whether a participant reported results of HIVST (Table 2), predictors of positive HIVST results among those who reported their results (Table 3), and predictors of linking to HIV care among persons who had positive HIVST results (Table 4). Persons who received a test kit at a place other than the workplace, mobile van and venue based were more likely to report their HIVST results [AOR 3.58 95%CI (1.30–14.84), *p* = 0.033]. Those with a secondary level of education or above [AOR 1.34 95%CI (1.00–1.78), *p* = 0.046] and those who had moderate alcohol use (1–6 drinks in the past week) [AOR 1.59 95%CI (1.28–1.99), *p* = < 0.001] were also more likely to report their results.

**Table 2:** Characteristics of participants receiving an HIVST kit who reported HIVST results.

	**Univariate**					**Multivariate**		
**Predictors**	***N***	**(%)**	**OR**	**(95% CI)**	***p* value**	**aOR**	**(95% CI)**	***p* value**
**Recruitment strategy**								
Mobile Van	1,881/2,344	(80%)	-ref-					
Venue- based	118/154	(77%)	0.81	(0.55–1.20)	0.277	1.04	(0.69–1.58)	0.863
Workplace	61/86	(71%)	0.60	(0.38–0.98)	0.036^*^	0.68	(0.42–1.15)	0.137
Other	47/50	(94%)	3.86	(1.41–15.93)	0.024	3.58	(1.30–14.84)	0.033
**Kit type**								
OraQuick	821/1,004	(82%)	-ref-			-ref-		
Atomo	1,286/1,630	(79%)	0.83	(0.68–1.02)	0.073	0.84	(0.68–1.03)	0.103
**Age**								
> 25	910/1,133	(80%)	-ref-			-ref-		
25–34	778/980	(79%)	0.94	(0.76–1.17)	0.595	1.03	(0.82–1.29)	0.793
≥35	419/521	(80%)	1.01	(0.78–1.31)	0.960	1.32	(0.97–1.80)	0.078
**Gender**								
Male	2,072/2,591	(80%)	-ref-			-ref-		
Female	35/43	(81%)	1.10	(0.53–2.55)	0.817	1.40	(0.66–3.32)	0.407
**Education**								
Primary	287/387	(74%)	-ref-			-ref-		
≥Secondary	1,810/2,231	(81%)	1.50	(1.16–1.92)	0.002^*^	1.34	(1.00–1.78)	0.046
**Marital status**								
Married	57/76	(75%)	-ref-			-ref-		
Not married	2,050/2,558	(80%)	1.35	(0.77–2.24)	0.271	1.30	(0.71–2.28)	0.376
**Number of current sex partners**								
1	1,087/1,368	(79%)	-ref-			-ref-		
0	66/90	(73%)	0.71	(0.44–1.18)	0.168	0.90	(0.55–1.53)	0.695
≥2	946/1,165	(81%)	1.12	(0.92–1.36)	0.272	1.03	(0.84–1.27)	0.757
**Ever tested for HIV**								
Yes	268/356	(75%)	-ref-			-ref-		
No	1,832/227	(81%)	1.37	(1.05–1.78)	0.018^*^	1.22	(0.92–1.61)	0.170
**Circumcised**								
No	1,034/1,318	(78%)	-ref-			-ref-		
Yes	1,034/1,266	(82%)	1.22	(1.01–1.49)	0.041^*^	1.17	(0.95–1.44)	0.147
**Alcohol use (drinks in past week)**								
0	847/1,100	(77%)	-ref-			-ref-		
1	1,024/120	(85%)	1.66	(1.35–2.05)	<0.001^*^	1.59	(1.28–1.99)	<0.001
≥7	220/303	(73%)	0.79	(0.59–1.06)	0.113	0.75	(0.56–1.02)	0.066

* *Odds ratios with p < 0.05.*

*Each AOR is adjusted for all potential predictors*.

**Table 3:** Predictors of a positive result among persons reporting results.

	**Univariate**					**Multivariate**		
**Predictors**	***N***	**(%)**	**OR**	**(95% CI)**	***p* value**	**aOR**	**(95% CI)**	***p* value**
**Recruitment strategy**								
Mobile van	133/1,881	(7%)	-ref-			-ref-		
Venue- based	19/118	(16%)	2.52	(1.46–4.16)	<0.001^*^	1.94	(1.02–3.50)	0.034
Workplace	4/60	(7%)	0.94	(0.28–2.33)	0.904	0.75	(0.22–1.96)	0.602
Other	1/47	(2%)	0.29	(0.02–1.32)	0.217	0.24	(0.01–1.19)	0.172
**Kit type**								
OraQuick	63/821	(8%)	-ref-			-ref-		
Atomo	94/1,285	(7%)	0.95	(0.68–1.33)	0.760	0.88	(0.62–1.26)	0.494
**Age**								
<25	27/910	(3%)	-ref-			-ref-		
25–34	85/777	(11%)	4.02	(2.61–6.37)	<0.001^*^	3.59	(2.28–5.82)	<0.001
≥35	45/419	(11%)	3.93	(2.42–6.51)	<0.001^*^	3.09	(1.79–5.40)	<0.001
**Gender**								
Male	2,072/2,591	(80%)	-ref-			-ref-		
Female	4/35	(11%)	1.62	(0.48–4.15)	0.371	1.37	(0.39–3.77)	0.582
**Education**								
Primary	34/287	(12%)	-ref-			-ref-		
≥Secondary	120/1,809	(7%)	0.53	(0.36–0.80)	0.002^*^	0.91	(0.57–1.46)	0.676
**Marital status**								
Married	4/57	(7%)	-ref-			-ref-		
Not married	153/2,049	(7%)	1.07	(0.43–3.57)	0.899	1.79	(0.66–6.33)	0.300
**Number of current sex partners**								
1	79/1,086	(7%)	-ref-			-ref-		
0	4/66	(6%)	0.82	(0.25–2.06)	0.712	1.06	(0.30–2.81)	0.919
≥2	72/946	(8%)	1.05	(0.75–1.46)	0.773	1.29	(0.91–1.84)	0.158
**Ever tested for HIV**								
No	35/268	(13%)	-ref-			-ref-		
Yes	122/1,831	(7%)	0.48	(0.32–0.72)	<0.001^*^	0.58	(0.38–0.91)	0.015
**Circumcised**								
No	112/1,033	(11%)	-ref-			-ref-		
Yes	41/1,034	(4%)	0.34	(0.32–0.72)	<0.001^*^	0.49	(0.33–0.72)	<0.001
**Alcohol use (drinks in 1 past week)**								
0	62/846	(7%)	-ref-			-ref-		
1	58/1,024	(6%)	0.76	(0.52–1.10)	0.145	0.73	(0.49–1.10)	0.128
≥7	34/220	(15%)	2.31	(1.46–3.60)	<0.001^*^	2.00	(1.22–3.24)	0.005

* *Odds ratios with p < 0.05.*

*Each AOR is adjusted for all potential predictors*.

**Table 4:** Predicators of linkage to care among persons with a positive HIVST result.

	**Univariate**					**Multivariate**		
**Predictors**	***N***	**(%)**	**OR**	**(95% CI)**	***p* value**	**aOR**	**(95% CI)**	***p* value**
**Recruitment strategy**								
Mobile van	86/128	(67%)	-ref-			-ref-		
Venue- based	12/19	(63%)	0.84	(0.31–2.39)	0.728	1.39	(0.33–6.67)	0.664
Workplace	4/4	(100%)	-	-	-	-	-	-
Other	0/1	(0%)	-	-	-	-	-	-
**Kit type**								
OraQuick	42/62	(68%)	-ref-			-ref-		
Atomo	60/90	(67%)	0.95	(0.47–1.89)	0.890	1.36	(0.59–3.14)	0.467
**Age**								
<25	14/25	(56%)	-ref-			-ref-		
25–34	56/82	(68%)	1.69	(0.67–4.24)	0.261	2.02	(0.67–6.13)	0.208
≥35	32/45	(71%)	1.93	0.70–5.42	0.205	2.73	(0.79–9.98)	0.118
**Gender**								
Male	99/148	(67%)	-ref-			-ref-		
Female	3/4	(75%)	1.48	(0.18–30.44)	0.735	0.58	(0.04–15.42)	0.700
**Education**								
Primary	21/33	(64%)	-ref-			-ref-		
≥Secondary	79/116	(68%)	1.22	(0.53–2.71)	0.630	2.08	(0.66–6.71)	0.210
**Marital status**								
Married	4/4	(100%)	-	-	-	-	-	-
Not married	98/148	(66%)	-	-	-	-	-	-
**Number of current sex partners**								
1	50/78	(64%)	-ref-			-ref-		
0	1/4	(25%)	0.19	(0.01–1.54)	0.154	0.09	(0.00–0.95)	0.068
≥2	50/68	(74%)	1.56	(0.77–3.20)	0.223	1.56	(0.72–3.49)	0.266
**Ever tested for HIV**								
No	27/34	(79%)	-ref-			-ref-		
Yes	75/118	(64%)	0.45	(0.17–1.08)	0.088	0.19	(0.05–0.60)	0.009
**Circumcised**								
No	75/110	(68%)	-ref-			-ref-		
Yes	24/38	(63%)	0.80	(0.37–1.76)	0.571	0.72	(0.29–1.80)	0.475
**Alcohol use (drinks in past week)**								
0	41/59	(69%)	-ref-			-ref-		
1	38/57	(67%)	0.88	(0.40–1.92)	0.744	0.89	(0.35–2.25)	0.810
≥7	22/33	(67%)	0.88	(0.35–2.22)	0.780	0.89	(0.31–2.54)	0.820

^*^Odds ratios with p < 0.05.

*Each AOR is adjusted for all potential predictors*.

Factors associated with an increased likelihood of a positive result were testing at venue based recruitment points [AOR 1.94 95%CI (1.02–3.50), *p* = 0.034], being between the ages of 25 to 34 years [AOR 3.59 95%CI (2.28–5.82), *p* = < 0.001],being 35 years or older [AOR 3.09 95%CI (1.79–5.40), *p* = < 0.001] and heavy alcohol use (more than seven drinks in the past week) [AOR 2.00 95%CI (1.22–3.24), *p* = 0.005]. Factors which were associated with a reduced risk of a positive result included previously testing for HIV [AOR 0.58 95%CI (0.38–0.91), *p* = 0.015) and being circumcised [AOR 0.49 95%CI (0.33–0.72), *p* = < 0.001].

Those who reported a positive HIVST result and had previously tested for HIV were less likely to link to care [AOR 0.19 95%CI (0.05–0.60), *p* = 0.009].

From those who reported their test results and completed post-test questionnaires, (*n* = 2,107), [1,875(89%)] said the reason for testing using HIVST was wanting to know their HIV status. One thousand nine hundred and seventeen (91%) said using the test kits was either easy or very easy, while [2,044(97%)] said that they would recommend HIVST to someone else. One thousand three hundred and twenty-seven (63%) said that they would pay for a kit if it was available in their communities.

There were seven participants who reported a positive last HIV test before taking an HIVST kit. From these, four reported a positive HIVST result, one reported a negative HIVST result, one did not use the kit, and one did not report their result.

In our second review of linkage to care 15 months after the end of the follow up period, of the 50 participants who reported a positive HIVST result but had no evidence of linkage to care at the end of the follow up period, [8(16%)] persons were identified in the NHLS database with evidence of engaging in HIV care. Thus, by 15 months after use of the HIVST, 110/157 (70%) persons with positive HIVST results had linked to care.

In a 4 month period, 56 HIV positive men were enrolled into DO ART through HIVST thereby increasing the proportion of DO ART enrolees who were men at the uMkhanyakude district site to [206(53%)] by the end of the study.

## Discussion

Large scale distribution of HIVST kits targeting men in rural northern KwaZulu-Natal was found to be feasible, acceptable, and effective at reaching men who have not tested and those below the age of 35. Fourteen percent of those who took HIVST kits reported to have never tested for HIV, 98% were men, and of those, 80% were below the age of 35. These results support findings by Johnson et al., where it was found that willingness to self-test by Zimbabwean men was high at around 85% (21). These results are also consistent with other multiple reports that have suggested that HIVST can increase uptake of testing among high risk groups that are under-represented in HIV testing programs (22–27). Our data provides further demonstration that HIVST is a promising strategy to increase testing uptake among hard-to-reach groups such as men in South Africa and could help to achieve the “first 95%” in the UNAIDS testing and care cascade by 2,030 (28).

HIVST successfully increased the proportion of men enrolled in the DO ART study from 47 to 53% in the uMkhanyakude district site thus highlighting the far-reaching effects that HIVST has in increasing HIV testing amongst men.

The HIVST blood-based test kit was found to be the preferred test over the oral HIVST kit; 62% of participants selected to use the blood-based HIVST kit. These results contrast with findings by Ritchwood et al. (29) which found that participants overwhelmingly preferred the oral based HIVST kits. That study was conducted among both male and female participants from a South African rural study setting. Findings by Tonen-Wolyec et al. also found that preference for oral based tests was greater than that of blood-based tests from both male and female participants in Kinshasa and Kindu in the Democratic Republic of Congo (30). Findings by Lippaman et al. found a similar preference for blood-based HIVST among South African men who have sex with men (31). Different preferences for oral and blood-based HIVST in diverse settings in Africa indicate that it is important to provide a choice between oral and blood-based tests when offering HIVST kits.

A total of 157 participants tested positive, of whom 102/130 (78%) linked to care within 7.4 months of testing. This linkage percentage is higher than that reported by other HIVST studies (22, 26) even though by the end of the study, 42/152 (28%) of those who tested positive had not linked to care. Additional work needs to be done to understand reasons for not linking to care, as the UNAIDS testing and care cascade goal of 95% of HIV positive people started on ART and 95% of those on ART achieving viral suppression will be not be achieved without additional interventions to support linkage among persons who learn that they are HIV positive through HIVST. We found evidence of linkage 15 months after the end of the follow up period in only [8(16%)] of 50 participants who had not linked to care at the end of the study follow-up, which highlights the ongoing barriers men face in accessing facility-based care such as inconvenient clinic hours and transportation problems, as previously reported (11). Community-based ART initiation has the potential to overcome these barriers as highlighted by Barnabas et al. (18) where it was found that community-based ART increased viral suppression rates amongst men. Community-based ART has the potential to increase viral suppression amongst men through immediate ART initiation within the community vs. delayed linkage through facility-based ART initiation which has shown to present a lesser percentage of those who eventually link to care over an extended period.

Factors associated with a reduced risk of a positive HIVST result included having previously tested for HIV and being circumcised. These results show that a greater amount of awareness and health education must be done on men who remain uncircumcised. It also highlights the importance of finding first time testers as these were found to be more likely to test positive. HIV self-testing has been proven to have the potential of reaching first time HIV testers in those hard-to-reach target groups such as men.

No emergencies were reported through the emergency number, supporting that an HIV positive result is no longer seen as a catastrophic psychological blow in this community. These results are consistent with the findings of Choko et al. (22), who found that there were no suicides or partner violence reported from those who tested positive through HIVST in a study conducted in 14 urban neighborhoods in Malawi with 6,124 male and 7,868 female participants, providing further evidence that expansion of HIVST does not lead to an increase in social harms in the community.

Out of seven individuals who indicated that they were aware of a previous positive test but were not on ART, one HIVST result came back negative highlighting the possibility of a false negative HIVST result. Although self-testing is associated with a high specificity, the tests can produce a small number of false negatives (32) but the possibility of the individual being on ART at the time of testing cannot be ruled out.

For scale-up of similar programs in different settings, we recommend that future implementers use strategies that we used in the areas which fell beyond the AHRI study area. In these areas, we first approached community leaders to gain permission to distribute kits. Thereafter, we approached the local clinics to make them aware of the work we were doing and to help them prepare for a possible increase in patients coming into the clinic for confirmatory testing and linkage to care. Thirdly, with assistance of the community leaders, we attended community meetings and gatherings where we made the community aware of the HIVST programme and encouraged them to join. Building a relationship with the community leaders was done in a timely manner without requiring massive additional resources, suggesting that these relationships could be recreated in other settings where they do not yet exist.

Our study had limitations, including that the proportion of first-time testers could be overestimated since some participants may have participated without disclosing that they already knew their HIV status to be positive. Health education on the inaccuracies of testing while on ART was given at every kit distribution event. Respondent social desirability bias could have influenced self-report of HIV testing history, HIVST results and linkage to care. Moreover, by the end of the second linkage review, more than a quarter of those who tested positive had not linked to care. This highlights two additional limitations. First, we did not have a confirmatory test result for people who did not link to care, thus we could be overestimating the number of people with HIV. Second, this is a limitation of the approach of engaging men into care because we cannot confirm the test results and progress on the cascade of those who did not link. A way to overcome this limitation is having community ART services readily available within the community to assist those who have tested positive to link to care. If these services are not easily accessible to men within the community, then the barriers to facility-based care which men experience (11) could prevent them from linking at a clinic.

Strengths of this study include showing that large scale distribution with over 900 HIVST kits per month with a focus on reaching men is both feasible and acceptable in this rural KwaZulu-Natal setting. The HIVST project was conducted with a maximum of 10 staff members with most distribution accomplished by the 4 recruiters. This is important because it shows that a high volume of kits can be distributed with a limited number of staff. This staff complement is similar to personnel available in many district settings highlighting that similar distributing strategies can be applied in such settings. Community health workers could also be trained to perform equivalent tasks in other settings. Another strength of the study was the success of the collaborative partnership between the community, community leaders and the local clinics. The study demonstrated valuable contributions of these stakeholders to the success of the HIVST programme.

## Conclusion

HIVST was effective in reaching younger men and those who were first time HIV testers in uMkhanyakude. As persons aged younger than 35 and men account for the highest percentage of persons who have never tested for HIV in KwaZulu- Natal (14), HIVST should be used as a component of strategies to reach this target population for testing in KwaZulu-Natal and South Africa at large. Additional interventions beyond HIVST are needed to support persons who test HIV positive with linkage to HIV care and ART.

## Data Availability Statement

The raw data supporting the conclusions of this article will be made available by the authors, without undue reservation.

## Ethics Statement

The studies involving human participants were reviewed and approved by Ethical approval was obtained from the University of Washington, Human Sciences Research Council and the University of KwaZulu-Natal Ethics Committees. The patients/participants provided their written informed consent to participate in this study.

## Author Contributions

AS, CC, and RB designed the study. NS, OK, and MK conducted the project and collected the data. NS, AS, and TS analyzed the data. NS and AS drafted the manuscript. All authors contributed to the revisions and content of the final manuscript.

## Conflict of Interest

The authors declare that the research was conducted in the absence of any commercial or financial relationships that could be construed as a potential conflict of interest.

## Publisher's Note

All claims expressed in this article are solely those of the authors and do not necessarily represent those of their affiliated organizations, or those of the publisher, the editors and the reviewers. Any product that may be evaluated in this article, or claim that may be made by its manufacturer, is not guaranteed or endorsed by the publisher.
